# Serotonin-Mediated Effects of Maternal Presence on Brain Activity in the Prefrontal Cortex of Pups: Implications for Maternal Neglect

**DOI:** 10.1523/ENEURO.0309-18.2018

**Published:** 2018-08-17

**Authors:** Rosalind S.E. Carney

**Highlighted Research Paper:** Maternal Regulation of Pups’ Cortical Activity: Role of Serotonergic Signaling, by, Emmanuelle Courtiol, Donald A. Wilson, Relish Shah, Regina M. Sullivan, and Catia M. Teixeira

Brain development in infants and children is severely impacted when bonding and attachment interactions with a primary caregiver do not occur. Such neglect includes the failure to provide basic needs, whether it be supervision and care, food, or hygiene (Robinson et al., 2017). In 2014, 7.1 per thousand children in the United States were victims of neglect (Child Trends, 2016). The perinatal period of brain development is particularly susceptible to these environmental insults. Neglect during this time can result in immediate abnormalities such as decreased brain electrical activity, decreased brain metabolism, and inadequate formation of reciprocal pathways between brain regions (National Scientific Council on the Developing Child, 2012). In the long-term, perinatal neglect can manifest in heightened fear, stress, depression, addiction, and cognitive defects (Nelson et al., 2011; Cameron et al., 2017). To mitigate these adverse consequences, it is crucial to ask: what are the chemical and physical substrates in the developing brain impacted by neglect?

Fortunately, this question arises amid a strong foundation of prior research that has examined the brain’s susceptibility to environmental insults. The prefrontal cortex (PFC) is particularly susceptible to developmental defects as its maturation period starts perinatally and extends through adolescence. The PFC contains multiple serotonergic receptors, including 5-HT2R. Modulation of serotonin (5-HT) signaling in the perinatal period can lead to abnormal emotional behavior in adult mice (Ansorge et al., 2004). Separating a dam from her pups serves as a model for replicating maternal absence in rats. A prior study used a wireless technique to record cortical low-field potentials (LFPs) in rat pups and showed that maternal presence was related to an increase in low-frequency neural activity (Sarro et al., 2014). In their *eNeuro* publication, Courtiol et al., 2018, used wireless recording technology to determine whether maternal absence affects PFC activity in rat pups and, if so, whether serotoninergic signaling contributes to this process.

Male rat pups, at postnatal day 9–12, were implanted with an electrode specifically targeted to the anterior cingulate cortex region of the PFC. The next day, wireless recordings of pup PFC activity were measured while the dam was present (IN) or absent (OUT) from the nest, the latter condition mimicking maternal neglect. These experiments showed that during the IN condition there was an overall tendency for increased PFC activity in pups. This indicates that maternal presence can modulate PFC activity in pups.

Next, the receptor for 5-HT2 (5HT2R) was inhibited by injecting pups with ketanserin. PFC activity was then wirelessly recorded as before during IN and OUT conditions. Ketanserin-treated pups did not exhibit the increase in PFC activity in response to maternal presence that had been previously observed. This finding was confirmed to be a specific effect of ketanserin by examining saline-injected controls and verifying the dams had similar levels of absence/presence compared to the prior experiment. These findings suggest that 5-HT2 signaling plays a significant role in the influence of maternal presence on PFC activity in pups.

Given the observed effects of ketanserin treatment, Courtiol and colleagues assessed how artificially enhancing 5-HT levels would affect PFC activity. Pups were injected with the selective serotonin reuptake inhibitor (SSRI) fluoxetine. To test the specificity of fluoxetine action on serotoninergic transmission, ketanserin was used to inhibit 5HT2Rs. For these experiments, pups were recorded in isolation, in a 500-ml plastic beaker that contained nest bedding. The findings showed that when pups were separated from the dam, serotonergic signaling was sufficient to increase PFC activity in pups and that this effect was mediated through 5HT2Rs.

Taken together, these findings shed light on the effect of serotonin on modulating PFC activity in pups with respect to maternal neglect. These data have the potential to provide insight into pathologic mechanisms, in particular serotonergic signaling defects, that lead to psychiatric illness as a result of neglect during infancy. Professor Teixeira’s group (New York University School of Medicine) hopes to inactivate serotonergic pathways for several days during the perinatal period and examine the consequences in adult rats. Hopefully, these analyses would lead to better treatments for individuals that experienced chronic neglect by primary caregivers, such as individuals who were in orphanages as children.

**Figure 1.**
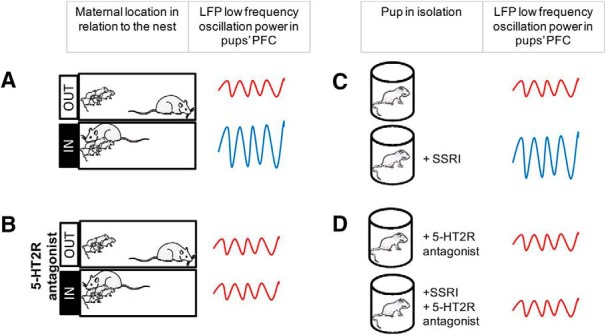
***A***, Maternal presence (IN) increases low-frequency oscillation power in the PFC of pups, when compared to maternal absence (OUT). ***B***, A 5-HT2R antagonist blocks the maternal regulation of pup PFC activity. ***C***, Administration of an SSRI increases PFC activity of pups in isolation compared to saline-treated controls. ***D***, This effect was blocked by a 5-HT2R antagonist. Blocking 5-HT2Rs while coadministering an SSRI confirmed involvement of 5-HT2Rs in mediating pup PFC activity.

## References

[B1] Ansorge MS, Zhou M, Lira A, Hen R, Gingrich JA (2004) Early-life blockade of the 5-HT transporter alters emotional behavior in adult mice. Science 306:879–881. 10.1126/science.1101678 15514160

[B2] Cameron JL, Eagleson KL, Fox NA, Hensch TK, Levitt P (2017) Social origins of developmental risk for mental and physical illness. J Neurosci 37:10783–10791. 10.1523/JNEUROSCI.1822-17.2017 29118206 PMC5678010

[B3] Child Trends (2016) Child maltreatment. Appendix 2. Last updated September, 2016. Retrieved from Available at https://www.childtrends.org/indicators/child-maltreatment.

[B8] Courtiol E, Wilson DA, Shah R, Sullivan RM, Teixeira CM (2018) Maternal Regulation of Pups' Cortical Activity: Role of Serotonergic Signaling. eNeuro 4:ENEURO.0093-18.2018. 10.1523/ENEURO.0093-18.2018 PMC6071199 30073196

[B4] National Scientific Council on the Developing Child (2012). The science of neglect: the persistent absence of responsive care disrupts the developing brain (Working Paper 12). Available at http://developingchild.harvard.edu/index.php/resources/reports_and_working_papers/working_papers/wp12/.

[B5] Nelson CA 3rd, Bos K, Gunnar MR, Sonuga-Barke EJ (2011) The neurobiological toll of early human deprivation. Monogr Soc Res Child Dev 76:127–146. 10.1111/j.1540-5834.2011.00630.x 25018565 PMC4088355

[B6] Robinson L, Segal J, Smith M (2017) Child abuse and neglect. How to spot the signs and make a difference. Last updated April, 2018. Available at https://www.helpguide.org/articles/abuse/child-abuse-and-neglect.htm.

[B7] Sarro EC, Wilson DA, Sullivan RM (2014) Maternal regulation of infant brain state. Curr Biol 24:1664–1669. 10.1016/j.cub.2014.06.017 24980504 PMC4108557

